# Mutation of SlARC6 leads to tissue-specific defects in chloroplast development in tomato

**DOI:** 10.1038/s41438-021-00567-2

**Published:** 2021-06-01

**Authors:** Jiang Chang, Fanyu Zhang, Haiyang Qin, Peng Liu, Jianfeng Wang, Shuang Wu

**Affiliations:** College of Horticulture, FAFU-UCR Joint Center and Fujian Provincial Key Laboratory of Haixia Applied Plant Systems Biology, Fujian Agriculture and Forestry University, Fuzhou, 350002 China

**Keywords:** Non-model organisms, Mutagenesis

## Abstract

The proliferation and development of chloroplasts are important for maintaining the normal chloroplast population in plant tissues. Most studies have focused on chloroplast maintenance in leaves. In this study, we identified a spontaneous mutation in a tomato mutant named *suffulta* (*su*), in which the stems appeared albinic while the leaves remained normal. Map-based cloning showed that *Su* encodes a DnaJ heat shock protein that is a homolog of the *Arabidopsis* gene *AtARC6*, which is involved in chloroplast division. Knockdown and knockout of *SlARC6* in wild-type tomato inhibit chloroplast division, indicating the conserved function of SlARC6. In *su* mutants, most mesophyll cells contain only one or two giant chloroplasts, while no chloroplasts are visible in 60% of stem cells, resulting in the albinic phenotype. Compared with mature tissues, the meristem of *su* mutants suggested that chloroplasts could partially divide in meristematic cells, suggesting the existence of an alternative mechanism in those dividing cells. Interestingly, the adaxial petiole cells of *su* mutants contain more chloroplasts than the abaxial cells. In addition, prolonged lighting can partially rescue the albinic phenotypes in *su* mutants, implying that light may promote SlACR6-independent chloroplast development. Our results verify the role of SlACR6 in chloroplast division in tomato and uncover the tissue-specific regulation of chloroplast development.

## Introduction

Chloroplasts are an important organelle where plants absorb solar energy and produce sugars^1^. The number, size, and morphology of chloroplasts directly affect leaf color and photosynthesis intensity.

Chloroplast division and proliferation are important for maintaining the chloroplast population. In *Arabidopsis*, a number of mutants that are defective in the accumulation and replication of chloroplasts (*arc*) have been identified, in which chloroplast number, size, and shape are severely affected^2–5^. Similar to their original microbial ancestors, chloroplasts replicate by binary fission in plants, which is driven by ring-like dynamic division machinery located at the middle of the organelle^6–9^. In plants, the contractile component of the division machinery is composed of the FtsZ ring (Z ring), which is located in the inner membrane of the chloroplast, and tubulin-like heteropolymer-forming proteins (FtsZ1, FtsZ2, and DRP5B), which are located in the outer membrane of the chloroplast^10–18^. *ARC6* and *PARC6* (paralog of *ARC6*) encode chloroplast-targeted proteins that assemble and stabilize the Z ring by directly interacting with FtsZ2 and FtsZ1^19–22^. ARC6 is closely related to Ftn2, a prokaryotic cell division protein. *PARC6* is unique in vascular plants. It is possible that *PARC6* was duplicated from *ARC6* after the separation between nonvascular and vascular plants^23^. In *Arabidopsis arc6* mutants, a mesophyll cell usually contains only two giant chloroplasts^2,21^. The chloroplast number in *parc6* mutants is tenfold less than that in the wild type (WT), while *PARC6* overexpression often inhibits chloroplast division by repressing FtsZ assembly^23–26^. ARC6 and PARC6 can recruit PLASTID DIVISION1 (PDV1) and PDV2, both of which are located in the outer envelope membrane (OEM)^23,27^. PDV1 and PDV2 then further recruit dynamin-related protein 5B (DRP5B/ARC5) to the OEM of the mid-chloroplast^28^. The Z ring is confined to the mid-chloroplast, and the formation of the Z ring requires the chloroplast Min system, which includes ARC3, MinD1, and MinE1^9^^,24,27,29–32^. Multiple chloroplast division site 1 (MCD1) is another plant-specific protein that is required for Z ring positioning^33,34^. It was previously shown that MCD1 interacted with ARC6 in the stroma and interacted with FtsZ2 in an ARC6-dependent manner^33^.

Much of our understanding of chloroplast division has been derived from studies on *Arabidopsis* leaves. However, fossil records revealed that most ancient vascular plants had only the axis without leaves, suggesting that chloroplasts emerged much earlier than leaves^35,36^. Thus, knowledge of chloroplasts derived from other tissues can contribute to our understanding of chloroplast division and physiology. In this study, we analyzed a tomato mutant named *su*, which is a naturally spontaneous mutant collected by the TGRC Tomato Genetics Resource Center (http://tgrc.ucdavis.edu). A prominent phenotype of *su* mutants is albinic stems with visually normal leaves. Bulked segregant analysis (BSA), map-based cloning, and functional verification by virus-induced gene silencing (VIGS) and CRISPR all showed that mutation of *SlARC6* led to the differential albinic phenotype. Further observations indicated that the chloroplasts in the stem of *su* mutants almost disappeared, while they fused into the giant abnormal plastids in leaves, suggesting the differential effect of *SlARC6* mutation on stems and leaves. The defective chloroplasts in the stem of *su* mutants could partially be rescued by prolonged light exposure, suggesting that light signaling could regulate chloroplast development.

## Results

### Phenotypic analyses of the *suffulta (su)* mutant

**Fig. 1:**
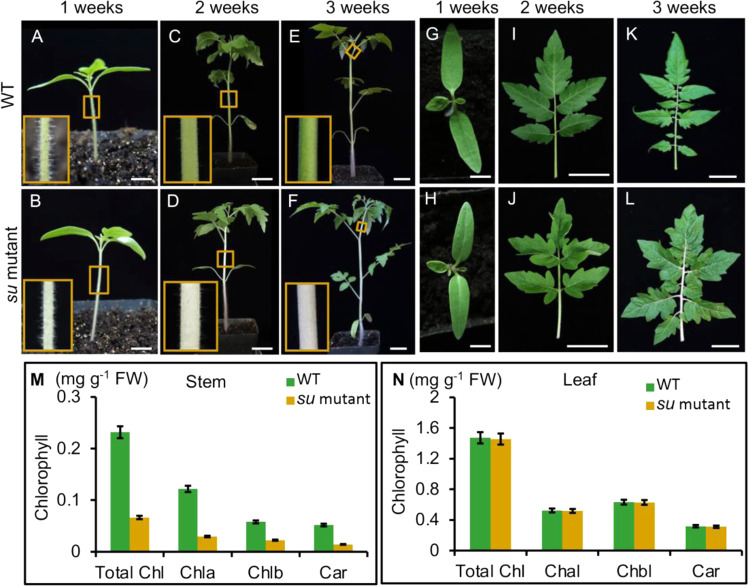
The phenotype of *su* mutants at the seedling stage. **A**, **B** The hypocotyl of WT and *su* mutants; bars: 0.5 cm. **C**–**F** The stems of WT and *su* mutants (2- and 3-week stages); bars: 3 cm. **G**, **H** The cotyledons and young leaves of WT and *su* mutants; bars: 0.5 cm. **I**–**L** The leaves of WT and *su* mutants (2- and 3-week stages). The lower right insets in **A**–**F** are magnified views of the corresponding boxed regions. Bars: 3 cm. **M**, **N** Comparison of chlorophyll contents in the stems and leaves of the WT and *su* mutant at the 3-week stage

In the natural lines collected by the TGRC (http://tgrc.ucdavis.edu/), we characterized a mutant named *su* (LA0628), in which the spontaneous mutation causes the phenotype of albinic stems. We first examined the color changes of stems and leaves over different developmental stages in *su* mutants and WT (Ailsa Craig) plants (Fig. 1A–L, Supplementary Fig. 1). In 1-week-old seedlings, *su* mutants exhibited pale hypocotyls, while the color of cotyledons and young leaves stayed similar to those in WT (Fig. 1A, B, G, H). At 2–3 weeks, the newly formed tissues, including the stems, petioles, and rachis, in *su* mutants were still albinic, but the leaves appeared WT-like (Fig. 1C–F, I–L). This phenotype persisted until the flowering and fruiting stages (Supplementary Fig. 1), suggesting that the defect of albinic stems is not a developmental stage-associated phenotype.

To further assess whether the albinic phenotype is related to chlorophyll, we measured the chlorophyll content in 3-week-old tomato seedlings. Our results showed that the levels of multiple chlorophylls, including Chla, Chlb, Car, and total Chl, in *su* stems were significantly lower than those in WT stems, but such a difference was not detected in the leaves (Fig. 1M, N). The pale color and the defective chlorophyll content prompted us to hypothesize that the albinic phenotype in *su* mutants might derive from the absence of chlorophyll precursors or dysfunctional chloroplasts.

### Fine mapping of *Su*

**Fig. 2:**
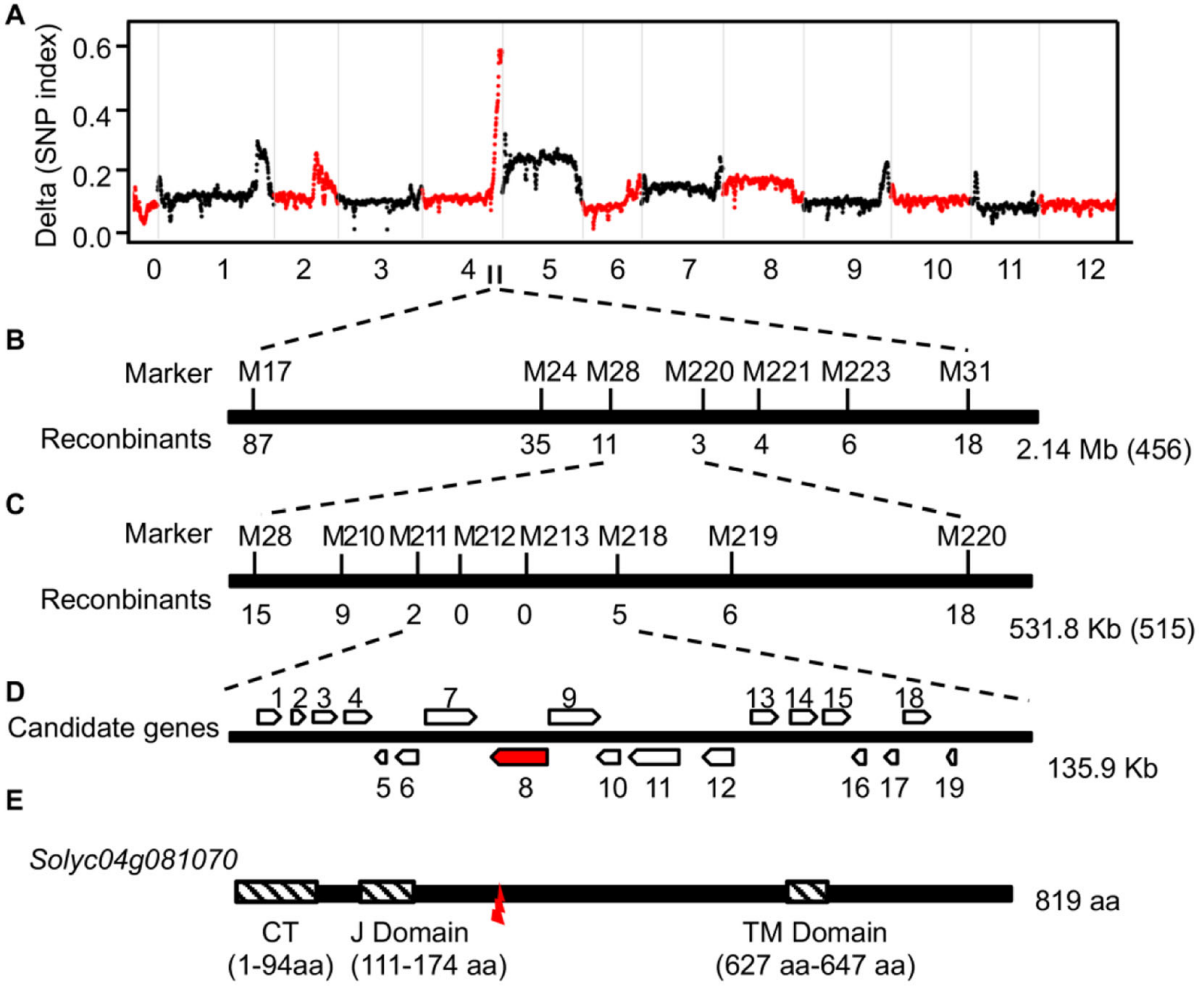
Map-based cloning of *Su*. **A** Mapping of *Su* by BSA-seq analysis. Significantly associated single-nucleotide polymorphisms (SNPs) were found between 63 and 66 Mb of chromosome 4. **B** Rough mapping of *Su*. The black line represents chromosome 4; characters above the line show markers used in the rough mapping; characters behind the line show the number of recombinations. The interval between markers M17 and M31 is 2.14 Mb. A total of 456 individuals with albinic stems were used in the rough mapping. **C** Fine mapping of *Su*. The interval between markers M28 and M220 is 531.8 Kb. A total of 515 individuals with white stems were used in the fine mapping. **D** Candidate genes in the target region. Boxes with arrows represent open reading frames (ORFs) in the target region according to the tomato genome (SL2.4). ORFs above the line are located in the positive strand of the chromosome, and ORFs below the line are in the antisense strand. The red box with arrow shows the target gene. **E** Gene structure of *ORF8*. The black line shows the coding sequence of *ORF8*, which encodes 819 amino acids. The hatched boxes represent domains of ORF8. The red lightning bolt shows the mutation site of *ORF8* in LA0628

To identify the mutation, we developed an F_2_ population by crossing *su* mutants to WT. All F_1_ plants exhibited normal color in both stems and leaves, indicating that the albinic phenotype was caused by a recessive mutation. In the F_2_ population, the separation ratio between normal stems and albinic stems was ~3:1, consistent with Mendel’s law of single-gene inheritance (Supplementary Table 1). Using a next-generation sequencing-based BSA approach, we identified an associated locus on the long arm of chromosome 4 (Fig. 2A). We next generated seven InDel molecular markers (M17, M24, M28, M220, M221, M223, and M31) in the candidate interval of 2 Mb between 63.5 and 65.5 Mb of chromosome 4. Using these InDel markers among 456 F_2_ plants, we delimited the *su* mutation to the region between markers M28 and M220, at which 11 and 3 recombination events were detected, respectively (Fig. 2B; Supplementary Table 2). There were ~30 candidate genes within this 320 kb region between M28 and M220. We thus developed six markers between M28 and M220 and further narrowed the candidate gene to the region between M211 and M218 at which only two and five recombination events were detected (Fig. 2C; Supplementary Table 2). The candidate gene seemed to cosegregate with markers M212 and M213 based on our observation in 551 F_2_ plants by the albinic stem (Fig. 2C; Supplementary Table 2). There were 19 putative ORFs (ORFs 1–19) in the 135 kb region between M211 and M218 (Fig. 2D; Supplementary Table 3).

We next amplified and sequenced the genomic sequences (including all introns and exons) of the 19 candidate genes. A comparative sequence alignment showed that there was a missense mutation with a C–T at 718 bp downstream of the predicted translation initiation site in ORF8 of *su* mutants, and the mutation site was heterozygous in F1 plants (Fig. 2E; Supplementary Fig. 2). According to the annotated tomato genome (ITAG release 2.4), ORF8 encodes a heat shock protein of 819 amino acids. The N-terminal region of ORF8 contains a putative DnaJ domain and a chloroplast-targeting signal. The C-terminal region contains a transmembrane domain (TMD). The mutation in *su* mutants led to a premature stop codon (TGA) in the truncated protein with a deletion of the conserved TMD domain (Fig. 2E). Phylogenetic analyses showed that the homolog of ORF8 in *Arabidopsis* is AtARC6, which was reported to function in chloroplast division (Supplementary Fig. 3)^37^. Together with the physiological phenotypes, we speculated that the mutation in s*u* mutants could cause defective division and development of chloroplasts.

### Functional verification of *Su* mutation

**Fig. 3:**
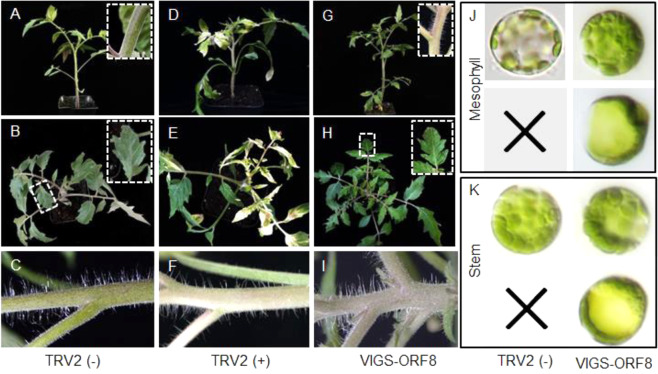
Functional verification of ORF8 by virus-induced gene silencing (VIGS). **A**–**I** The phenotype of VIGS-ORF8 plants. **A**–**C** Plants injected with *Agrobacterium* containing empty TRV2 were used as the negative control. **D**–**F** VIGS-PDS plants were used as the positive control. **G**–**I** The VIGS-ORF8 plants. **J** Protoplasts from the mesophyll of the negative plants (the left channel) and VIGS-ORF8 plants (the right channel). **K** Protoplasts from the stem of the negative plants (the left channel) and VIGS-ORF8 plants (the right channel). The panels filled with crosses show that there are no undivided chloroplasts in the negative plants. Both undivided and divided chloroplasts could be found in the stems of VIGS-ORF8 plants

To further test ORF8 function in tomato, we knocked down ORF8 by VIGS. To this end, a fragment of ORF8 was inserted into the pTRV2 vector for infection. We used phytoene desaturase (PDS) in the same vector as the positive control, and empty pTRV2 was used as the negative control. The WT-like appearance of the negative control (Fig. 3A–C) and photobleaching phenotype in the positive control (Fig. 3D–F) indicated the effectiveness of VIGS of PDS. As expected, ORF8 silencing showed albinic stems without a leaf phenotype, which was similar to that in *su* mutants (Fig. 3G–I). Consistent with the phenotype, the expression level of ORF8 was markedly decreased in both leaves and stems (Supplementary Fig. 4).

**Fig. 4:**
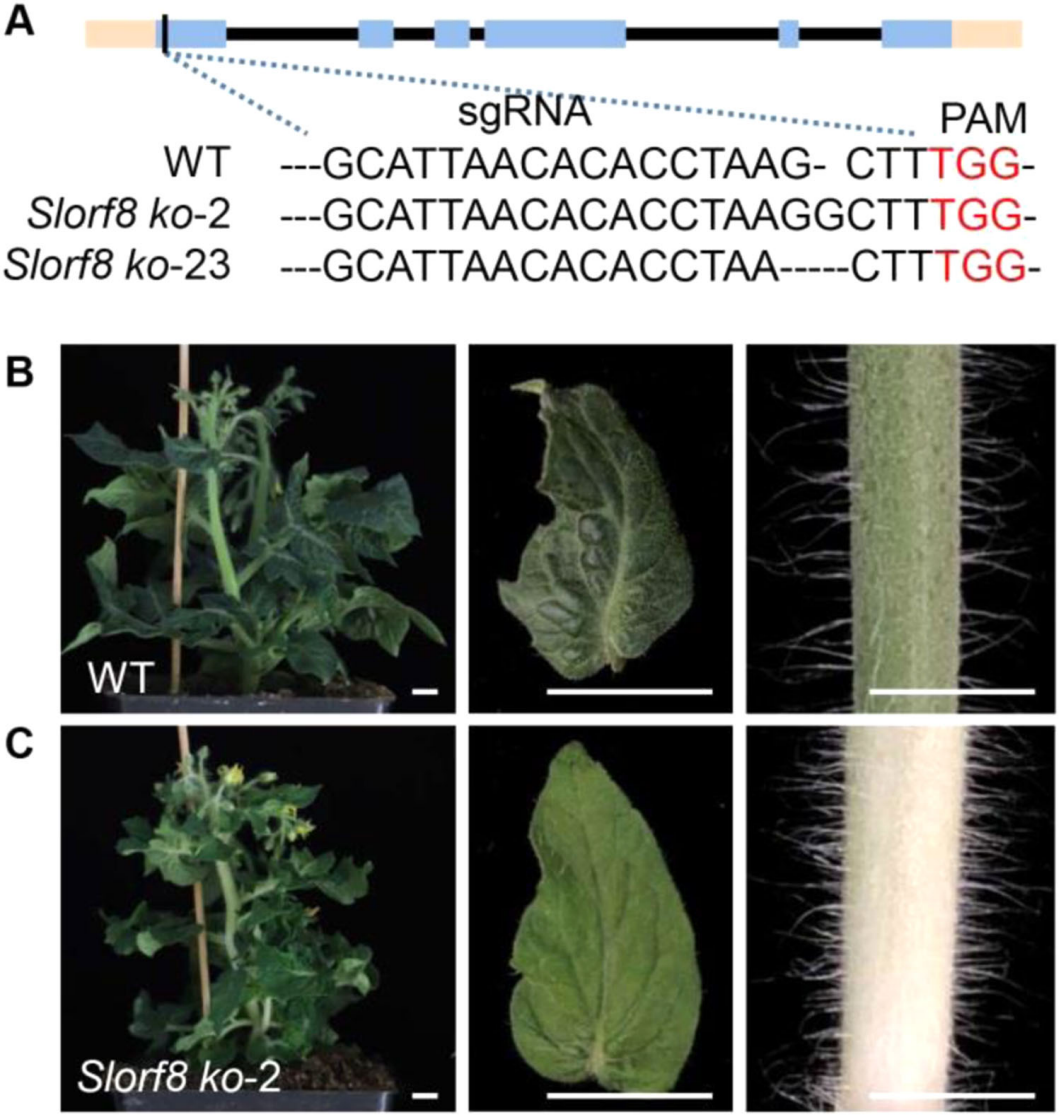
Functional verification of ORF8 by CRISPR. **A** *Slorf8* edited alleles found in the transgenic plants. Blue boxes represent exons, black boxes represent introns, and pink boxes represent UTR regions. The sgRNA and PAM sequence are shown. Two homozygous edited lines were used in this study, one showing a G insertion and one a G deletion. **B** Wild-type (WT) plants were used as the negative control. **C** The phenotype of the *Slorf8* ko-2 line. Bars: 1 cm

To examine whether ORF8 functions in chloroplast division, we isolated mesophyll protoplasts from leaves and the epidermis of stems. Under microscopy, we observed many protoplasts with only one (or occasionally a number of) giant chloroplast in the ORF8 VIGS-silenced plants, which was in sharp contrast with multiple round chloroplasts in the protoplasts isolated from the negative control (Fig. 3J, K). To further verify this result, we constructed *orf8* mutants using the CRISPR/Cas9 technique (Fig. 4). Two homozygous CRISPR lines were obtained: line 2 with a G insertion and line 23 with a G deletion (Fig. 4A). Similar to the *su* mutants, the ORF8 CRISPR lines showed albinic stems (Fig. 4A, C). These results indicate that ORF8 is indeed the target gene.

### Tissue specificity of chloroplast development in tomato

**Fig. 5:**
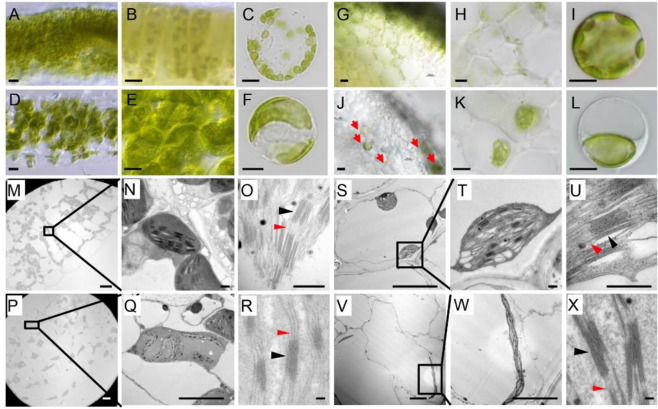
Visualization of the chloroplast and thylakoid. The chloroplast phenotype in the leaves of WT (**A**–**C**) and *su* mutants (**D**–**F**) under DIC. Cross-sections of the leaves of WT (**A**) and *su* mutants (**D**); cross-sections of the palisade tissues of WT (**B**) and *su* mutants (**E**); mesophyll protoplasts from WT (**C**) and *su* mutants (**F**). Bars: 10 μm. The chloroplast phenotype in the stem of WT (**G**–**I**) and *su* mutants (**J**–**L**) under DIC. Cross-sections of the stem of WT (**G**, **H**) and *su* mutants (**J**, **K**). Stem protoplasts from WT (**I**) and *su* mutants (**L**). The red arrows mark cells containing chloroplasts. Bars: 10 μm. The ultrastructure of chloroplasts from the leaves of WT (**M**–**O**) and *su* mutants (**P**–**R**). The ultrastructure of chloroplasts from the stems of WT (**S**–**U**) and *su* mutants (**V**–**X**). Black and red arrowheads mark granum and stromal thylakoids, respectively. Bars:10 μm (**M**, **P**, **S**, **V**); bars: 500 nm (**N**, **O**, **Q**, **R**, **T**, **U**, **W**, **X**)

Loss of function of ARC6 in *Arabidopsis* was shown to disrupt the stabilization of the Z ring during chloroplast division^21,22^. However, it is unclear why the albinic phenotype was only observed in the stem of *su* mutants. Interestingly, in the tissue sections prepared by the vibratome, we indeed observed a remarkable alteration of chloroplast morphology in the leaves of *su* mutants. The mesophyll cells of the *su* mutants usually contained only one or two giant chloroplasts, which was apparently different from the multiple round-shaped chloroplasts in the WT mesophyll cells (Fig. 5A–F). In contrast to the case of the leaves, the cross-sections of the *su* stems showed that the chloroplasts almost all disappeared (Fig. 5G–J). Occasionally, a number of cells containing one or two giant chloroplasts were observed in stem cells, and those chloroplasts were ~396 μm^2^ (area) in size, which is 40 times that of the WT (Fig. 5K, L; Supplementary Fig. 5). To further confirm this observation, we performed high-resolution imaging by transmission electron microscopy (TEM). The overall morphology and the number of chloroplasts in *su* mutants were consistent with the observation of the live tissue sections (Fig. 5M, P, S, V). In WT, the ultrastructure of chloroplasts appeared uniform, with clearly visible stacks of thylakoids (Fig. 5N, Q, T, W). However, the ultrastructure of chloroplasts in *su* mutants was fairly diverse under TEM (Fig. 5O, R, U, X). The lamellae comprising grana thylakoids and stroma thylakoids were sparse in *su* mutants (Fig. 5O, R, U, X). These results suggest that the absence of chloroplasts, as well as the sparse thylakoids and stromal thylakoids in the giant chloroplasts, contribute to the albinic stem phenotype in *su* mutants.

### The role of SlARC6 in chloroplast division in meristematic cells

**Fig. 6:**
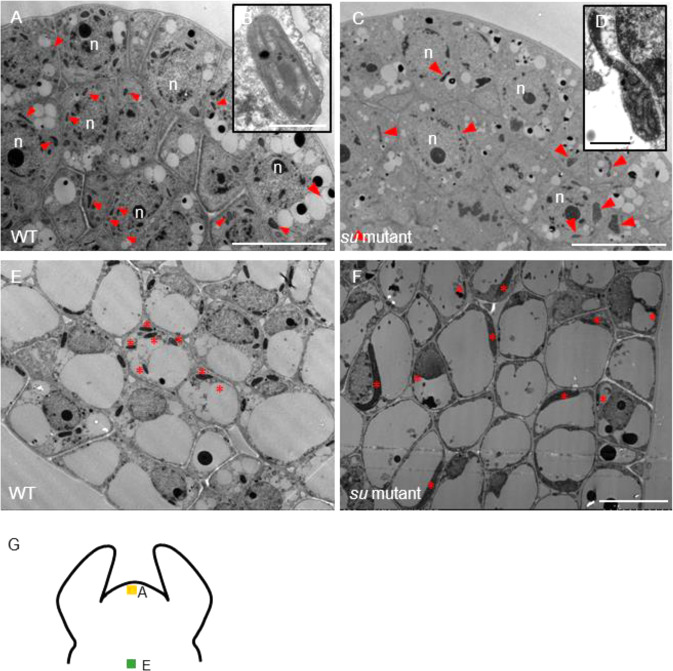
TEM observation of the chloroplasts in the meristem. TEM micrographs showing longitudinal sections of the shoot apical meristem in WT (**A**, **B**) and *su* mutants (**C**, **D**). **B**, **D** The upper right insets show the chloroplast in the shoot apical meristem (bar: 1 μm). n nucleus. The red triangles point to the proplastids and chloroplasts. Bar: 10 μm. **E**, **F** TEM micrographs showing the transverse section of the region beneath the meristem. The red stars mark the chloroplast. Bar: 10 μm. **G** Schematic showing the position of TEM observations in the meristem. The yellow square shows the position of images in **A**–**D**. The green square shows the position of images in **E**, **F**

One prominent difference between the leaf and the stem is the cell division pattern during postembryonic growth. Leaf growth involves continuous cell division and cell expansion, while stem cells mostly undergo cell expansion. To understand whether cell division is the major reason for the distinct chloroplast phenotype, we examined meristematic cells. The typical meristem dome consists of three cell layers, in which most cells contain several immature vacuoles^38^. Chloroplasts are derived from proplastids, which feature two envelope membranes and limited stromal thylakoids^3,39^. In the meristem, proplastids and chloroplasts were found in almost all cells in both WT and *su* mutants, but some chloroplasts of *su* mutants also showed abnormal morphology (Fig. 6A–D). It is possible that chloroplast division and cell division are tightly associated. We next observed the lower part of the meristem where cell division is as rare as in the stem (as shown in Fig. 6G). The cells in this area featured large but not fully expanded vacuoles (Fig. 6E, F). Compared with WT cells, many cells in this area of *su* mutants contained abnormal chloroplasts (Fig. 6E, F). Compared with those in the meristem, most chloroplasts in mature stems disappeared, suggesting that the chloroplasts may divide concomitantly with cell division but do not divide when there is only cell expansion in the stem.

### Light can partially rescue the albinic phenotype

**Fig. 7:**
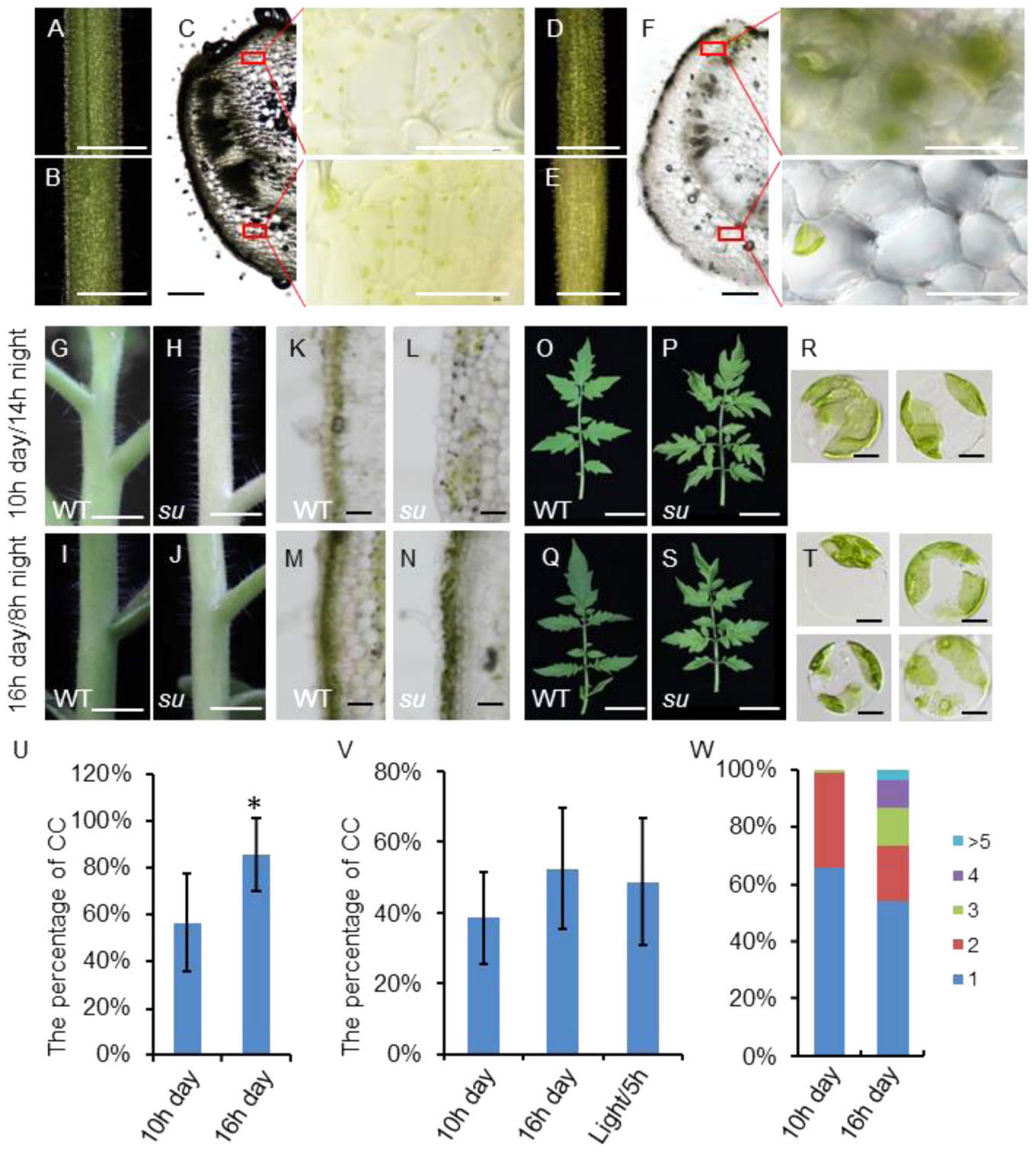
Light can increase the chloroplast number in *su* mutants. The adaxial (**A**) and abaxial (**B**) surfaces of the petioles of WT. Bar: 0.5 cm. **C** The cross-section of WT petiole. The magnified views represent the adaxial and abaxial surfaces of the WT petiole. Bar (**C**): 1 mm. The adaxial (**D**) and abaxial (**E**) surfaces of the *su* mutant petiole. Bar: 0.5 cm. **F** The cross-section of the petiole in *su* mutants. The magnified views represent the adaxial and abaxial surfaces of the petiole in *su* mutants. Bar (**C**): 1 mm. Stems of WT and *su* mutants grown under conditions of 10 h day/14 h night (**G**–**J**) or 16 h day/8 h night (**K**–**N**). **I**–**J**, **M**–**N** Sections of the stem in WT and *su* mutants. Leaves of WT and *su* mutants grown under conditions of 10 h day/14 h night (**O**–**Q**) or 16 h day/8 h night (**R**–**T**). **Q** There were one or two giant chloroplasts in the leaf protoplasts from *su* mutants grown at 10 h day/14 h night. **T** There were one to four giant chloroplasts in each leaf protoplast from *su* mutants grown at 16 h days/8 h night. **U** Statistical analysis of the percentage of cells containing chloroplasts (CC) in the total epidermal cell population. The stems of *su* mutants grown under conditions of 10 h day/14 h night (10 h day) or 16 h day/8 h night (16 h day) were analyzed. The bars represent the standard deviation (SDs) of 50 biological replicates. The asterisk indicates a significant difference by t-test: *0.01 < *P* < 0.05. **V** Statistical analysis of the percentage of protoplasts containing chloroplasts (CC) in the total protoplast population. The stems of *su* mutants grown under conditions of 10 h day/14 h night (10 h day) or 16 h day/8 h night (16 h day) were used for protoplast isolation. In addition, some protoplasts from the stems of *su* mutants grown on a 10 h day/14 h night were exposed to light for an additional 5 h. The bars represent the standard deviation (SD) of five biological replicates. **W** Qualification of chloroplasts in protoplasts. The *x*-axis shows *su* mutants grown under lighting conditions of 10 h day/14 h night (10 h) or 16 h day/8 h night (16 h). The *y*-axis shows the proportion of chloroplasts in each protoplast. The blue column shows the proportion of protoplasts containing one chloroplast; the red column shows the proportion of protoplasts containing two chloroplasts; and so on. Fifty protoplasts were quantified for the treatment of 16 h days/8 h night, and eighty protoplasts were quantified for the treatment of 10 h day/14 h night

In the WT, the adaxial and abaxial surfaces of the petiole seemed to have no noticeable difference in color (Fig. 7A, B). However, in *su* mutants, compared with the adaxial surface, the abaxial surface appeared to be yellowish (Fig. 7D, E). We then observed the chloroplasts in the fresh tissue sections of the petiole. Under differential interference contrast (DIC) microscopy, the epidermal cells of the petiole in WT were full of chlorophyll, so the cells appeared to be opaque, whereas the epidermal cells in *su* mutants seemed to be transparent due to the lack of chloroplasts (Fig. 7C, F). Interestingly, the adaxial surface of *su* mutants was more transparent than the adaxial side. Under the microscope, the chloroplast number within the cells on the adaxial side was significantly higher than those on the abaxial surface in *su* petioles (Fig. 7C, F). This distinction could derive from the different light received by adaxial and abaxial sides.

Light plays an important role in chloroplast development and chlorophyll biosynthesis^40^^,^^41^. To assess the role of light in the albinic phenotype, we cultured both WT and *su* mutants under conditions of 10 h day/14 h night or 16 h day/8 h night for 20 days. The WT and *su* mutants grown under longer lighting had greener leaves and stems than those grown on the 10 h day/14 h night (Fig. 7G–J, O–S). The stems of *su* mutants seemed to have the most significant change in color (Fig. 7G–J). In the freshly sectioned tissues, we observed a substantial rise in chloroplast-containing cells in *su* stems after prolonged light exposure, with cell chloroplast-containing cells elevated from ~50% under 10 h day/14 h night conditions to ~90% under 16 h day/8 h night conditions (Fig. 7G–N, U). In the protoplasts isolated from the epidermal cells of stems, we found that the proportion of chloroplast-containing cells increased from 38 to 52% with prolonged light exposure, confirming that light could promote the formation of chloroplasts. To further verify this, we isolated protoplasts from the stems of *su* mutants grown under 10 h day/14 h night conditions and then exposed the protoplasts to light for 5 h. Our quantification indicated that the proportion of chloroplast-containing protoplasts increased from 38 to 48% (Fig. 7U). In addition, we quantified the average number of chloroplasts within the protoplasts of *su* mutants. The results showed that up to 99% of the *su* protoplasts contained one or two chloroplasts at 10 h day/14 h night, while ~27% of the protoplasts contained three or more chloroplasts at 16 h day/8 h night (Fig. 7O–S, V). This result indicates that light can promote chloroplast development in *su* mutants.

## Discussion

Chloroplasts are biological factories where plants transform solar energy into organic substances for plant growth and development. Thus, it is important to maintain the appropriate number and physiology of chloroplasts. In *Arabidopsis*, a number of mutants named *ARCs* were reported to have defective chloroplast division. In *Arabidopsis* mutants of *ARC* family members, larger chloroplasts in mesophyll cells were observed^2,4,5^. In *Arabidopsis*, *arc6* mutants have abnormal chloroplasts in mesophyll cells but no other dramatic changes in whole plants^21^. Here, we found that the mutation of *SlACR6* in tomato leads to the albinic phenotype in stems. A similar phenotype was previously reported in three *su* mutants that had slightly paler leaves and albinic stems^42^. Interestingly, the results here demonstrated phenotypic variability among the three accessions, implying that *su* mutations with different backgrounds could affect the phenotype^42^.

In higher plants, chloroplasts divide and replicate via a contractile division complex including the FtsZ ring (Z ring), a protein complex located on the stromal surface of the inner envelope membrane, and the ARC5 ring, a protein complex located on the cytosolic surface of the OEM^10–18^. ARC6 has evolved from a prokaryotic cell division protein and bears three conserved domains: the N-terminal region, which protrudes into the stroma and directly interacts with FtsZ2; the C-terminal region, which extends into the intermembrane space and interacts with PDV2; and a TMD^27^. The ARC6–FstZ1 interaction is required for the Z ring to localize to the stromal surface of the inner envelope membrane^23,24,27^. In *su* mutants, the absence of the TM domain and the C-terminal region of SlACR6 may result in failed localization on the inner membrane of chloroplasts, which also prevents the localization of the Z ring mid-chloroplast. However, the number of chloroplasts was dramatically different in the leaves and stems of *su* mutants. In mature mesophyll cells, chloroplasts inherited from mother cells propagate through chloroplast division. Previous observations in *Arabidopsis arc6* mutants showed that the cells in the shoot apical meristem, leaf primordium, and mature leaves all contain two larger plastids, suggesting that plastids could divide during cell division^2,21^^,^^37^. In the leaves of tomato *su* mutants, we found a similar phenotype but rarely observed any chloroplasts in stems, suggesting that chloroplast development was entirely blocked in expanding cells. Based on these findings, we speculated that the tissue specificity of plastid development is likely not caused by differential ACR6 functions but instead by the distinct cell division patterns between leaves and stems. The mutation of ACR6 provides a good example to gain insight into the spatial and tissue-specific regulation of chloroplast development.

In *Arabidopsis*, overexpression of PDV1 and PDV2 can increase the number of chloroplasts. Interestingly, expression of PDV1 and PDV2 can be promoted by exogenous cytokinin treatment or overexpression of cytokinin-responsive transcription factor 2^43^^,^^44^. In addition to cytokinin, GA-deficient mutants of *Arabidopsis* (*ga1-3*) and *Oryza sativa* (*d18-AD*) both exhibited reduced chloroplast division and decreased expression of FtsZ2, ARC6, DRP5B, and PDV^45^. As GA and cytokinin coordinate to regulate the development of stems and leaves from the shoot apical meristem, the varied distribution of these two hormones in different tissues could be involved in the tissue specificity of chloroplast division. In addition to hormones, FHY3, a key regulator of far-red light signaling, was reported to activate the expression of ARC5, and the large chloroplast phenotype in *fhy5* mutants could be rescued by expression of *ARC5*^46^^,^^47^. This result suggests that light could also be a key regulator of chloroplast division. The partial complementation of *su* mutants by extended lighting provides further evidence that light is involved in ARC-regulated chloroplast division.

## Materials and methods

### Plant materials and growth condition

Spontaneous *su* mutants LA0628 and LA1589 and Ailsa Craig (AC, accession number LA2838A) were provided by the Tomato Genetics Resource Center (http://tgrc.ucdavis.edu). The mapping population of *Su* was constructed by crossing the *su* mutant and LA1589. In addition, the F2 population was obtained from the F1 selfing. All plants were cultured in a greenhouse at 26 °C.

### Determine of chlorophyll content

A 0.2 g sample was placed in a 2 mL centrifuge tube with liquid nitrogen and then ground into powder. Chlorophyll was extracted in methanol, and the absorbance was detected using a microplate fluorometer at 666, 653, and 470 nm. The chlorophyll contents were calculated using the following formulas: Chl *a* (mg/g FW^−1^) = (15.65 × A_666_ − 7.34 × A_653_) × V/FW; Chl *b* (mg/g FW^−1^) = (27.05 × A_653_ − 11.21 × A_666_) × V/FW; and Car (mg/g FW^−1^): (1000 × A_470_ − 2.86 × Chl *a* − 129.2 × Chl *b*) × V/245FW^48^.

### Bulked segregant analysis

Individuals (>30) exhibiting the *su* mutant-like phenotype and individuals (>30) exhibiting the WT phenotype were collected from the F_2_ population generated by crossing the *su* mutant and LA1589. Their genomic DNA was extracted by CTAB. Two micrograms of DNA was mixed to construct two samples (mutant-like and WT samples). Genome sequencing of the two samples was performed by HiSeqXten-PE150 (Novogene, Beijing) with a depth of 30× coverage of the tomato genome. The candidate region was analyzed according to the method in ref. ^49^.

### Map-based cloning

InDel molecular markers were designed in the candidate region according to the resequenced DNA and were polymorphic between the *su* mutant and LA1589^50^ (Supplementary Table 3). First, seven markers were analyzed using 456 individuals exhibiting a *su* mutant-like phenotype of the F_2_ population. The candidate region was determined according to the recombinants. Then, six markers in the candidate region were analyzed using 515 individuals exhibiting a *su* mutant-like phenotype. Finally, the candidate gene was delimited in a smaller interval.

### Virus-induced gene silencing

A fragment of ARC6 was designed using the VIGS tool (https://vigs.solgenomics.net) and inserted into the pTRV2 vector (named TRV2-ARC6). A *PDS* gene was also inserted into the pTRV2 vector and used as the positive control. pTRV1, pTRV2, pTRV2-*PDS*, and pTRV2-ARC6 vectors were transferred into *Agrobacterium tumefaciens* (GV3010) and then grown on LB plates with 100 μg/mL kanamycin and 50 μg/mL rifampicin. When tomato plants had two fully expanded cotyledons, we injected the cotyledons with an *A. tumefaciens* suspension. The details for preparing the *A. tumefaciens* suspension and injection are the same as those in ref. ^51^. The plants were cultured at 24 °C in a growth chamber. After 20 days, the phenotype was recorded.

### Construction of knockout plants

To generate the CRISPR/Cas9-Slorf8 construct, we inserted two target sites of Slorf8 (http://skl.scau.edu.cn/targetdesign) into the pTX vector using the Clone Express II One Step Cloning Kit (Vazyme Biotech C112-01/02). The construct was introduced into tomato cv. Micro-Tom by *Agrobacterium* (*A. tumefaciens*) mediated transformation. Homozygous transgenic plants were used for phenotypic characterization.

### Protoplast isolation

Tomato leaves and stems were cut into strips and put into an enzyme solution (cellulose R10, 2%; macerozyme R10, 0.4%; mannitol, 0.6 M; KCl, 20 mM; MES, 20 mM; pH 5.7; after 10 min at 55 °C, added CaCl_2_, 0.2 M; bovine serum albumin, 0.1%) to be digested for 3 h at 28 °C in the dark. Then, the mixture was filtered with a 100 μm strainer. Subsequently, the solution was centrifuged at 100 × *g* for 3 min, and the supernatant was discarded. The deposit, containing chloroplasts, was washed twice with W5 solution (NaCl, 154 mM; CaCl2.2H2O, 18.4 g; KCl, 0.4 g, MES, 2 mM; pH 5.7) and observed by DIC microscopy.

### Vibratome sectioning

Agarose (5%) was boiled in a microwave oven, and the agarose solution was cooled to ~65 °C before being poured into a tube. Subsequently, a specimen was suspended in the agarose solution, and the air bubbles were aspirated. The tube was put at 4 °C for hardening. Specimen blocks were cut into 80 µm sections by a vibratome. The vibratome sections were observed by DIC microscopy

### Transmission electron microscopy

Fresh samples were cut into 1 mm × 3 mm strips and fixed in 2.5% glutaraldehyde overnight at 4 °C. The glutaraldehyde was removed, and the samples were washed three times with phosphate buffer (0.1 M, pH 7.0). The samples were fixed again in 1% (wt/vol) osmium tetroxide for 1.5 h at room temperature. Osmium tetroxide was removed, and the samples were washed three times with phosphate buffer (0.1 M, pH 7.0). After that, the samples were dehydrated by using successive treatments in 50, 70, 80, 90, 95, and 100% (all vol/vol) ethanol for 20 min and 100% (all vol/vol) acetone for 20 min. Then, the samples were embedded in Spurr’s low-viscosity resin. After polymerization for 24 h at 70 °C, the ultrathin sections were cut on an ultramicrotome with a diamond knife and picked up on copper grids. Before observation, the samples were stained with uranyl acetate and lead citrate. Images were captured using a Hitachi-7650 transmission electron microscope.

## Supplementary information

Supplementary information
