# Supplementary material for: Mutation of SlARC6 leads to tissue-specific defects in chloroplast development in tomato
Source: Hortic Res. 2021 Jun 1;8:127. doi: 10.1038/s41438-021-00567-2 (PMC8167136; doi:10.1038/s41438-021-00567-2)

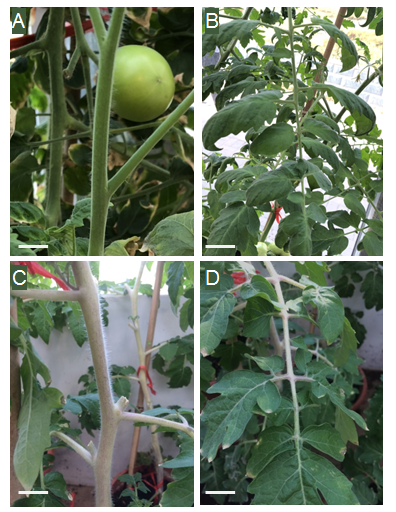


Supplementary Figure 1. Phenotypes of *su* mutants at the flowering and fruiting stages. (A, B) The phenotypes of the stem (A) and leaf (B) of WT. (C, D) The phenotypes of the stem (C) and leaf (D) of *su* mutants. Bar: 2 cm.


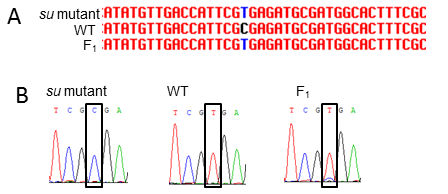


Supplementary Figure 2. Verification of the mutation of ORF8.

(A) The coding sequence of ORF8 in the *su* mutant, WT and F_1_ generation. There is a single-base substitution (C718T). (B) The peaks from the Sanger sequencing of ORF8 in the *su* mutant, LA1589 and F_1_ generation. The black boxes show the mutation site. Note the mutation is heterozygous in F1 plants.


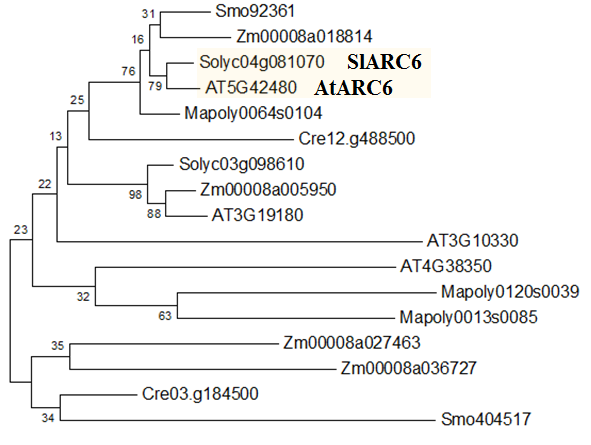


Supplementary Figure 3. Cladogram of homologous genes of SlARC6. The ID of SlARC6 is Solyc04g081070.


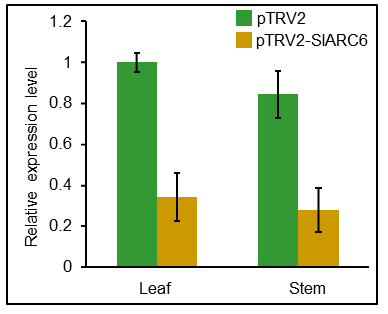


Supplementary Figure 4 The expression level of *SlARC6* in the leaves and stems of VIGS-SlARC6 plants.


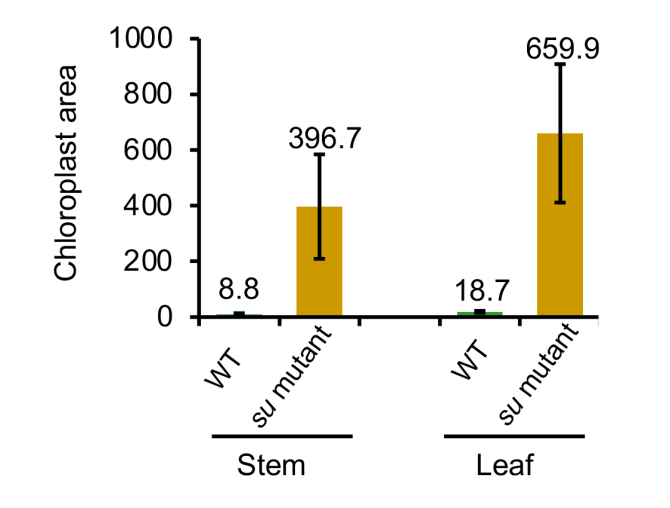


Supplementary Figure 5. Comparison of the chloroplast area in WT and *su* mutants.

Supplementary Table 1. *su* acts as a recessive gene.


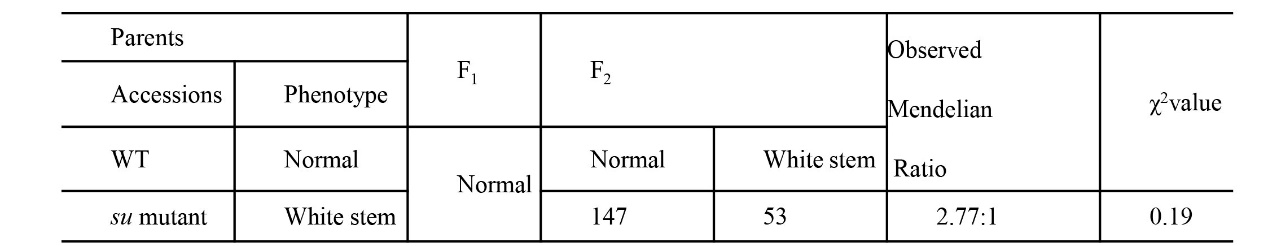


Supplementary Table 2. Markers used in map-based cloning.


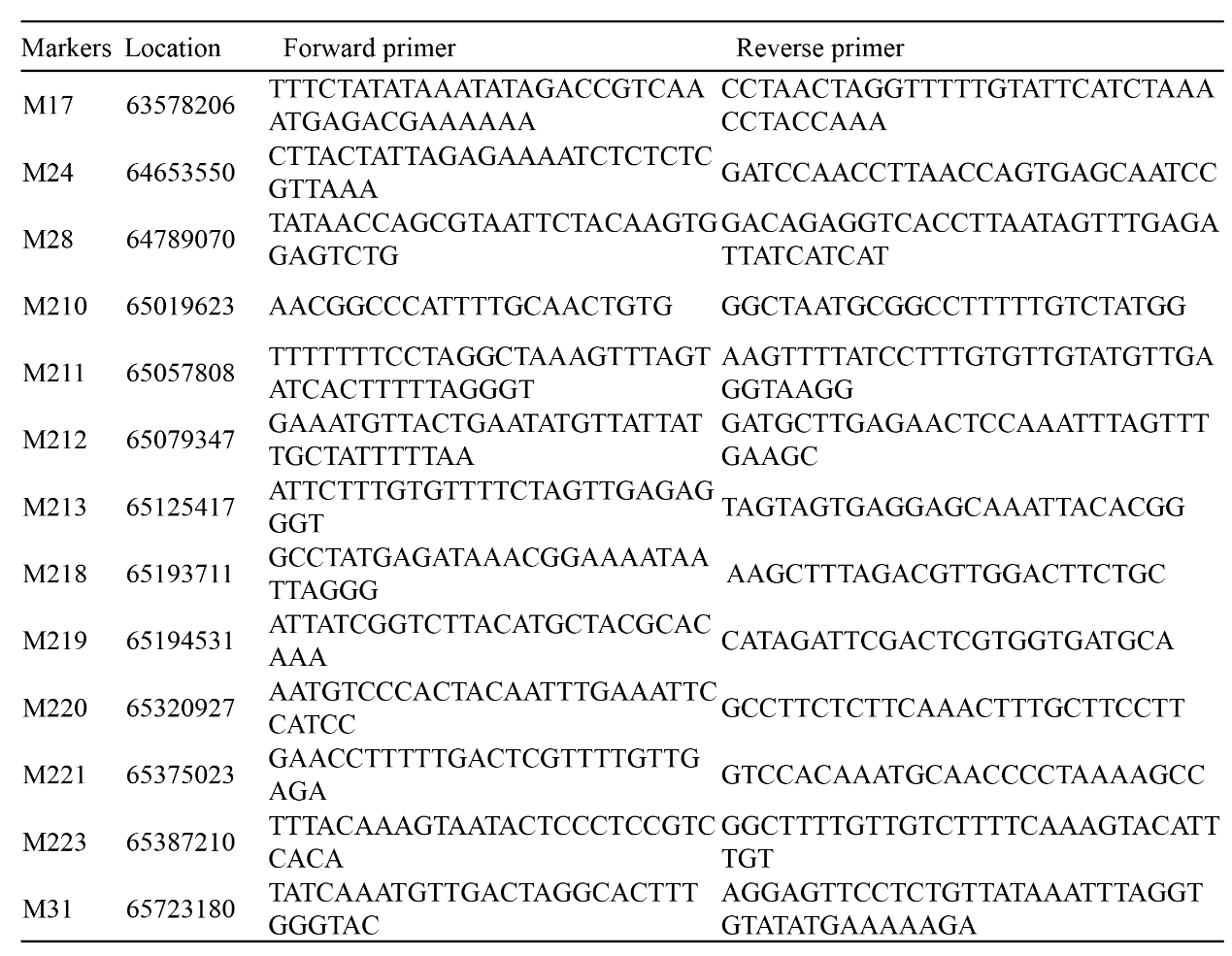


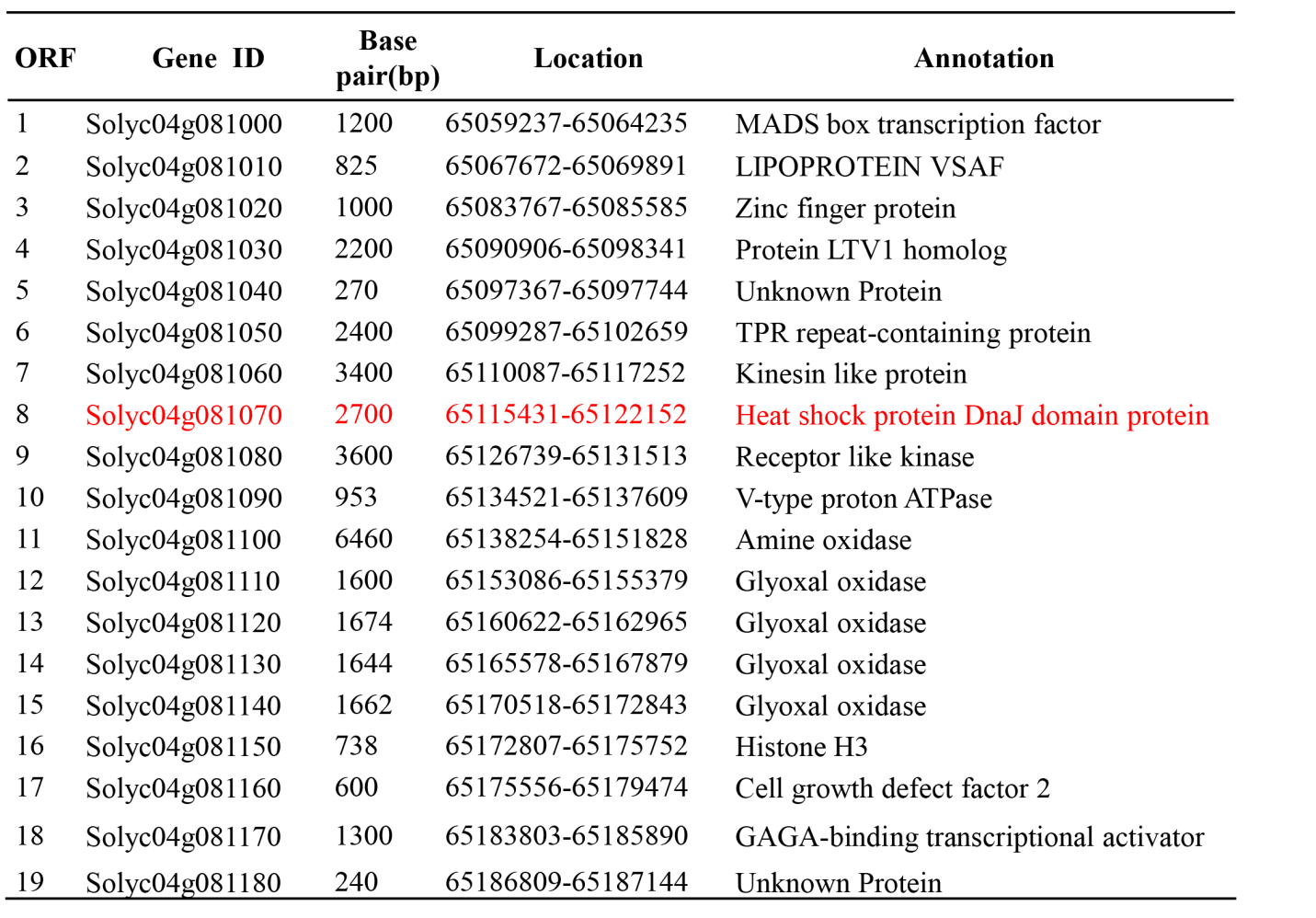

Supplement: Supplementary file 1 — Supplementary information [file 41438_2021_567_MOESM1_ESM.docx]
